# Transport mechanisms at the malaria parasite-host cell interface

**DOI:** 10.1371/journal.ppat.1009394

**Published:** 2021-04-01

**Authors:** Josh R. Beck, Chi-Min Ho

**Affiliations:** 1 Department of Biomedical Sciences, Iowa State University, Ames, Iowa, United States of America; 2 Department of Microbiology and Immunology, Vagelos College of Physicians and Surgeons, Columbia University, New York, New York, United States of America; Joan and Sanford I Weill Medical College of Cornell University, UNITED STATES

## Abstract

Obligate intracellular malaria parasites reside within a vacuolar compartment generated during invasion which is the principal interface between pathogen and host. To subvert their host cell and support their metabolism, these parasites coordinate a range of transport activities at this membrane interface that are critically important to parasite survival and virulence, including nutrient import, waste efflux, effector protein export, and uptake of host cell cytosol. Here, we review our current understanding of the transport mechanisms acting at the malaria parasite vacuole during the blood and liver-stages of development with a particular focus on recent advances in our understanding of effector protein translocation into the host cell by the *Plasmodium* Translocon of EXported proteins (PTEX) and small molecule transport by the PTEX membrane-spanning pore EXP2. Comparison to *Toxoplasma gondii* and other related apicomplexans is provided to highlight how similar and divergent mechanisms are employed to fulfill analogous transport activities.

## Introduction

Phylum Apicomplexa comprises a large group of obligate intracellular protozoan parasites that invade and subvert the cells of their hosts to create a niche for replication. These pathogens are an enduring burden in human and veterinary medicine and, in the case of malaria, have had a considerable impact on the human genome and the history of our species [1]. Life within the host intracellular milieu requires major investment to protect against host defenses and ensure a steady nutrient supply. As such, these parasites are superb manipulators of the biology of their host cells [2]. Most apicomplexan parasites reside within a parasitophorous vacuole (PV) during their intracellular life stages that is formed by invagination of the host cell plasma membrane during invasion [3–6]. The PV provides a protective environment but also necessitates coordination of several transport activities across this barrier to create and maintain the intracellular niche. Here, we review our current understanding of the transport mechanisms operating at the host–parasite interface of the blood-stage malaria parasite and provide a comparison to analogous activities at the liver-stage and in the related parasite *Toxoplasma gondii*.

**Fig 1:**
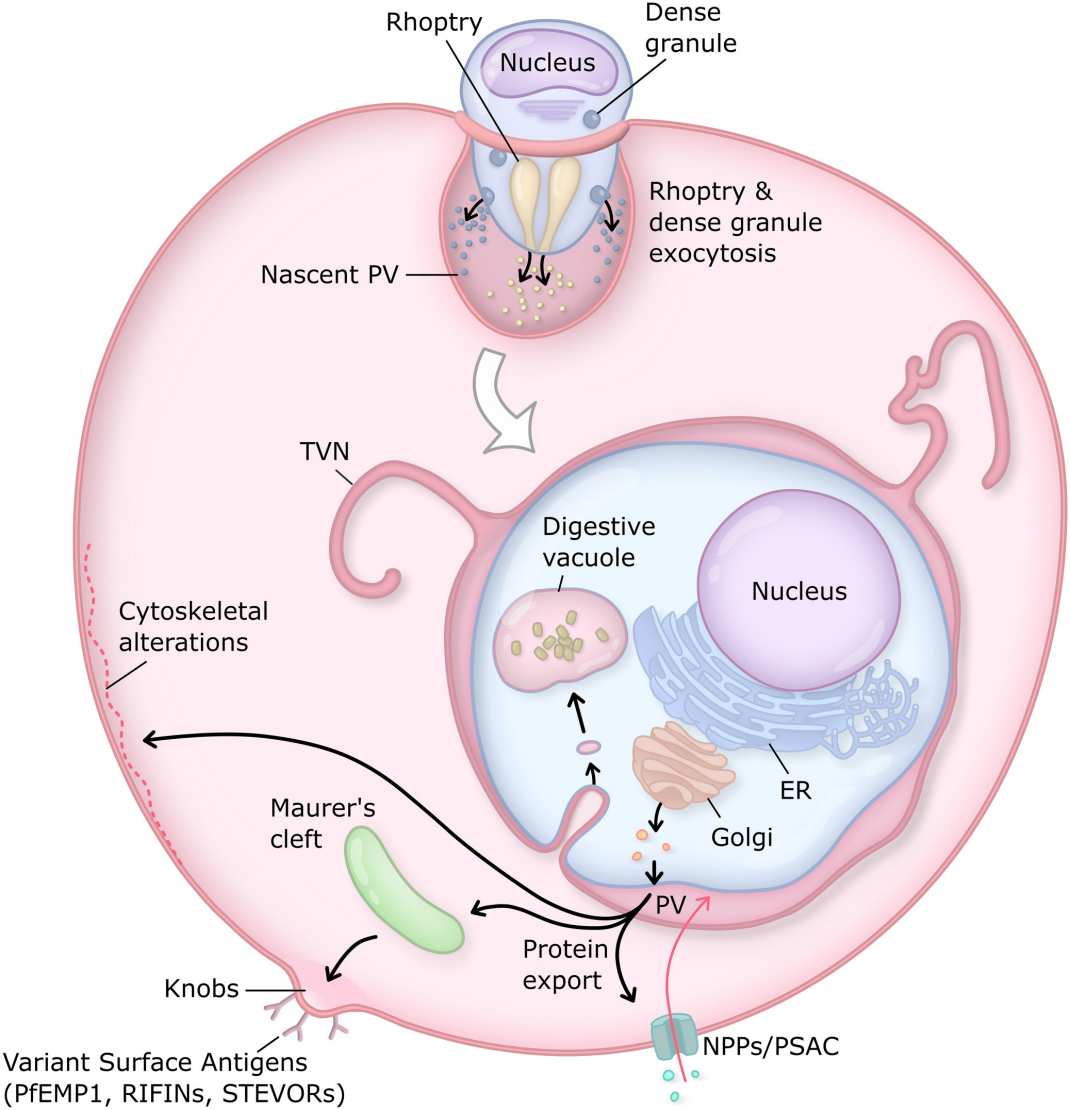
The *Plasmodium* intraerythrocytic PV. The PV is generated through invagination of the erythrocyte membrane during invasion. Discharge of the rhoptry and dense granule secretory organelles during and immediately after invasion rapidly establishes transport activities at the PVM. This is followed by a major wave of protein export during the early stages of intraerythrocytic development to remodel the host cell. During this time, the parasite exomembrane system is also generated, including TVN extensions of the PV thought to provide greater surface area for nutrient uptake and Maurer’s clefts, which serve as trafficking platforms for parasite exported effectors en route to the host membrane. Near the end of this period, a major increase in host membrane permeability is facilitated by activation of NPPs including PSAC. In addition to these changes in erythrocyte permeability, key host modifications include cytoskeletal rigidification and the formation of raised knobs on the erythrocyte surface for display of adhesins that mediate iRBC sequestration and immune evasion. In *P*. *falciparum*, the responsible variant surface antigens include PfEMP1, the RIFINs, and the STEVORs, each encoded by large, multigene families. Endocytic uptake of hemoglobin-rich host cytosol and degradation in the digestive vacuole liberates amino acids to support parasite metabolism and frees space for expansive growth within the erythrocyte. ER, endoplasmic reticulum; iRBC, infected red blood cell; NPPs, new permeability pathways; PfEMP1, *P*. *falciparum* erythrocyte membrane protein 1; PSAC, Plasmodial surface anion channel; PV, parasitophorous vacuole; PVM, PV membrane; RIFIN, repetitive interspersed family protein; STEVOR, sub-telomeric variable open reading frame protein; TVN, tubulovesicular network.

As mammalian erythrocytes are terminally differentiated and lack the wherewithal to support active growth and division, blood-stage malaria parasites export a large array of effector proteins that comprehensively remodel the host cell to create a hospitable intracellular environment [7–9]. These dramatic rearrangements to the erythrocyte enable the parasite to evade host defenses and provide the infrastructure necessary for protein and small molecule trafficking. In *Plasmodium falciparum*, the most virulent human malaria parasite, the effector repertoire is thought to contain >500 parasite proteins [8,10,11]. Many of these proteins are members of multigene families of membrane adhesins (*P*. *falciparum* erythrocyte membrane protein 1 [PfEMP1], repetitive interspersed family proteins [RIFINs], and sub-telomeric variable open reading frame proteins [STEVORs]) which are inserted into the erythrocyte plasma membrane where they facilitate adherence to the endothelial lining of capillaries, sequestering the infected red blood cell (iRBC) from circulation to evade splenic filtration [12]. Exported effectors also restructure the cytoskeleton and alter the permeability of the RBC to provide for nutrient acquisition and waste efflux [7–9]. An extensive exomembrane system, of which the PV membrane (PVM) is a key component, facilitates the trafficking of proteins between the intravacuolar parasite and the host cell surface, while endocytosis and catabolism of host cytosol opens space and supplies amino acids to enable parasite growth [5] (Fig 1).

## Protein export

### Traffic to the PV

The first parasite proteins to reach the forming vacuole are released from the apical rhoptry organelles during invasion, followed by a second secretion event from the spherical dense granules [13,14] (Fig 1). In *Plasmodium* spp., these secretory organelles disappear shortly after invasion until daughter merozoite formation during schizogony [15,16] with the PV serving as the default secretory destination of the developing intracellular parasite [17,18]. PV resident proteins contain an N-terminal signal peptide that is cleaved by signal peptidase upon endoplasmic reticulum (ER) entry, similar to default secretion in other eukaryotes. Although proteins destined for export into the erythrocyte also begin their journey by trafficking to the PV, the mechanisms governing their entry into and movement through the secretory pathway are more complex. Most exported proteins contain a *Plasmodium* EXport ELement (PEXEL, also known as the host-targeting signal), typically downstream of a recessed signal peptide [19,20] and appear to enter the ER through a distinct SEC translocon complex that includes the SEC61 channel and SPC25, a non-catalytic component of the signal peptidase complex [21]. Upon ER entry, the PEXEL sequence (typically RxLxE/Q/D, although some functional noncanonical PEXEL motifs have been identified [10,22]) is cleaved between the third and fourth residues by Plasmepsin 5, an aspartic protease which appears to operate in place of the classical signal peptidase SPC21, followed by acetylation of the newly exposed N-terminal residue [21,23–25]. While PEXEL processing is necessary for export of proteins bearing this motif, it is not strictly required as a number of PEXEL-negative exported proteins (PNEPs) are known which typically contain a signal peptide and transmembrane domain [26] and appear to enter the ER independent of SPC25 [21]. Despite these distinctions, the mature N-termini of PEXEL and PNEP proteins contain functionally equivalent information to mediate export [27], and these pathways converge at the PVM for translocation into the erythrocyte [28–30] by a process that requires ATP and protein unfolding [31,32].

### PVM translocation

**Fig 2:**
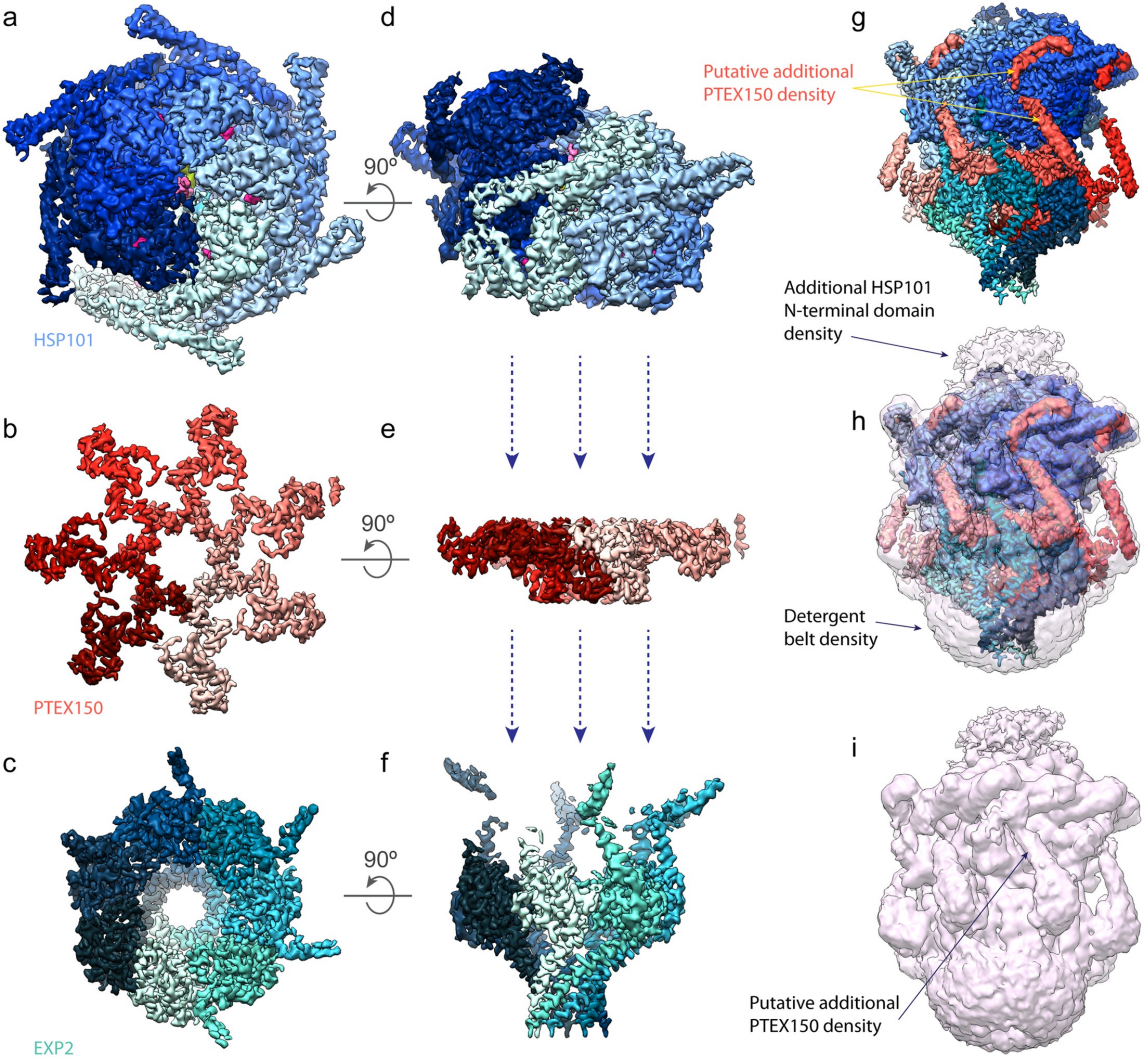
CryoEM structure of PTEX. Top and side views of the HSP101 (a, d), PTEX150 (b, e), and EXP2 (c, f) cryoEM maps are shown. Protomers of each protein are colored as increasingly dark gradients of blue (HSP101), salmon (PTEX150), or aquamarine (EXP2). Also visible in the HSP101 portion of the map are molecules of ATPγS (magenta) bound to each NBD as well as density corresponding to the polypeptide backbone of cargo within the HSP101 channel (pink). The cargo is engaged by NBD2 pore loops colored light blue (protomers 1–3) and yellow (protomers 4–6). The high-resolution cryoEM map of the full PTEX complex is shown (g), with the same color scheme as in panels (a–f). The same cryoEM map at a lower threshold is shown overlaid with the high-resolution cryoEM map (h) to display additional lower-resolution features, including an additional density above HSP101 which most likely corresponds to the HSP101 NTDs, which are not resolved in the high-resolution structure. Also visible is the detergent belt, as well as long helical densities extending from the PTEX150 (668–823) region. These densities appear to connect to additional densities on top of the HSP101 M-domains. The cryoEM map at a lower threshold is shown alone (i) to allow better visualization of the additional densities. cryoEM, cryo-electron microscopy; EXP2, exported protein 2; HSP101, heat shock protein 101; M-domains, Middle domains; NBD, nucleotide-binding domain; NTD, N-terminal domain; PTEX, *Plasmodium* Translocon of EXported proteins.

**Fig 3:**
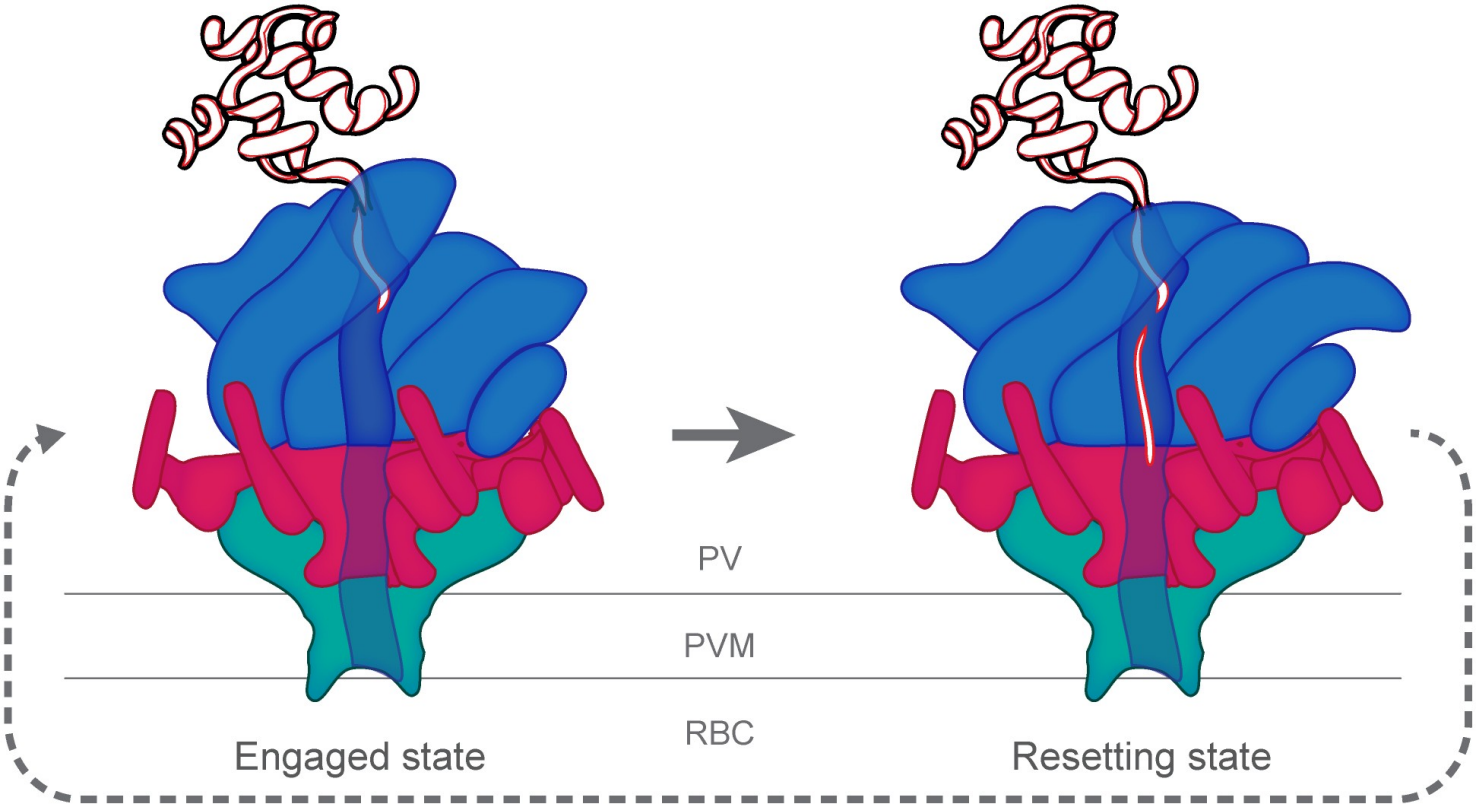
Mechanism of effector protein export by PTEX. Model for PVM translocation mechanism is shown based on the PTEX engaged and resetting states observed by cryoEM. Similar to other HSP100s, pore loops projecting from the HSP101 NBDs interact with cargo in the channel. Conformational changes in the HSP101 hexamer enable these loops to grasp successive portions of the cargo, pull it into the channel, and hold it in place to prevent backsliding. Repeated cycles mediate stepwise unfolding and threading of cargo through HSP101 and across the PVM via the PTEX150 flange-like adaptor and EXP2 membrane-spanning pore. cryoEM, cryo-electron microscopy; EXP2, exported protein 2; HSP101, heat shock protein 101; NBD, nucleotide-binding domain; PTEX, *Plasmodium* Translocon of EXported proteins; PVM, PV membrane; RBC, red blood cell.

Effector protein translocation across the PVM is carried out by the *Plasmodium* Translocon of EXported proteins (PTEX) [28,29,33–35], a 1.6-MDa membrane protein complex that consists of a protein-unfolding motor attached to a PVM-spanning pore [33] (Fig 2A–2G). This unique translocon contains 3 core components, the first of which is a hexamer of heat shock protein 101 (HSP101), an AAA+ ATPase chaperone most closely related to the Clp/HSP100 family of disaggregases [35]. Arranged in an asymmetric right-handed spiral, the HSP101 hexamer uses power derived from ATP hydrolysis to unfold effector proteins destined for export [33]. Unfolded cargo exiting the HSP101 oligomer is protected by a flange-shaped structure formed by a heptamer of PTEX150, which connects the base of the unfoldase to the funnel-shaped entrance of a membrane pore formed by 7 copies of exported protein 2 (EXP2) [33]. A comparison of the atomic models of PTEX in 2 distinct conformations obtained by single-particle cryo-electron microscopy (cryoEM) analysis of material purified directly from the endogenous source suggests a translocation model in which exported cargo is unfolded by HSP101 and fed through the PTEX150 adaptor and EXP2 PVM pore to reach the host cytosol (Fig 3) [33].

Several additional PV proteins are known to interact with PTEX but are here considered as accessory factors by virtue of their dispensability for the essential function of the PTEX core complex during intraerythrocytic development in culture. Accessory factors broadly conserved in *Plasmodium* spp. include thioredoxin 2 (TRX2) and PTEX88, identified together with the core complex [35], as well as the GPI-anchored surface protein Pf113 [36] and the Exported Protein Interacting Complex (EPIC) comprising parasitophorous vacuole protein 1 (PV1), PV2, and EXP3 [37]. These are mostly novel proteins not conserved outside of *Plasmodium* spp. making inference to specific functions difficult, although TRX2 is an active thioredoxin which has been suggested to aid in remodeling disulfide bonds to mediate unfolding of exported cargo or regulation of PTEX [29,38–40]. Additionally, although unrelated to known chaperones, PV1 has been proposed to assist PTEX as a co-chaperone based on its ability to replace HSP40 in a refolding chaperone system comprised of HSP70, HSP40, and the HSP101-related disaggregase HSP104 (all from *Chaetomium termophilum*) [41]. While these proteins can be disrupted or conditionally depleted during in vitro culture (most with little to no observable impact on protein export or parasite fitness), these accessory factors appear to aid in export of effector subsets that are important in the context of the vertebrate host, and some are crucial in vivo [29,37,40,42–44].

Compartmentalization by, and translocation across, membranes are innovations essential to the emergence and evolution of life. While SEC61/Y translocons are widely conserved [45], exclusively posttranslational protein translocation machines are highly diversified and generally more complex. For instance, bacterial translocons can rely on ABC transporters, trafficking ATPases, or proton gradients [46–48]. Some organellar protein import machines also employ proton gradients to power cargo transport [49,50], but most rely on chaperones acting on either or both sides of the membrane barrier [51,52]. In contrast to the complexity of these translocation machineries, PTEX is simpler yet more versatile, seemingly using HSP101 alone to unfold hundreds of structurally and functionally diverse proteins and thread them through the transmembrane pore formed by EXP2. HSP101 is also unique within the Clp/HSP100 family of AAA+ ATPases, as other members are generally protein unfoldases involved in protein quality control via disaggregation, disassembly, or proteolysis [53–57]. EXP2 family members appear to constitute an apicomplexan-specific PVM nutrient channel (see below) [58,59] that has been further adapted as a protein-conducting pore in blood-stage malaria parasites where PTEX150 represents the key innovation in the development of this novel translocon.

This unique *Plasmodium* solution to vacuolar translocation may reflect the special constraints of the erythrocyte host niche. In contrast to the extensive host machinery available to most intracellular pathogens for exploitation, mammalian erythrocytes have little existing infrastructure for the parasite to co-opt. As such, the modifications the parasite must make to its host cell are more extensive, requiring hundreds of proteins of diverse structure and function to comprehensively remodel the erythrocyte [8,9,60]. In the absence of cell autonomous immunity or an intact major histocompatibility complex (MHC) pathway, the intraerythrocytic malaria parasite can be less discriminating about the sheer quantity and variety of effector proteins delivered into the host compartment. Rather than employing multiple cargo-specific secretion systems like intravacuolar bacteria [46], the entire blood-stage malaria parasite exportome appears to thus be accommodated by a single highly versatile translocation machine. The relative simplicity of PTEX may also reflect a need to prepackage the system into dense granules so that it can be easily inserted into the PVM as it forms during invasion, allowing the parasite to begin translocating effectors into the erythrocyte immediately after invasion.

## Small molecule transport

### Soluble nutrient transport

Intraerythrocytic malaria parasites acquire a number of soluble nutrients from the serum, necessitating transport across several membranes to reach the parasite cytosol [61]. Among these are pantothenate for synthesis of coenzyme A, isoleucine for protein synthesis (the only amino acid not available in adult human hemoglobin, see “Host cytosol uptake” below), glucose to fuel glycolytic metabolism, and a variety of lipids [62,63]. Most solutes cross the erythrocyte membrane via new permeability pathways [64], the best defined being the Plasmodial surface anion channel (PSAC) [61,65], while glucose uptake appears to occur primarily through the Glut1 transporter [66]. Following entrance into the erythrocyte cytosol, the PVM must then be crossed to access transporters at the parasite plasma membrane (PPM). A key insight into the PVM transport mechanisms involved was provided by “PV-attached” patch-clamp studies in blood-stage *P*. *falciparum* parasites which identified a nutrient-permeable channel subsequently shown to possess a size cutoff of approximately 1,400 Da [67,68]. Strikingly, studies in the related apicomplexan *T*. *gondii* revealed free diffusion of dyes up to approximately 1,300 Da between the host cytosol and PV lumen, suggesting that a similar passive pore in the PVM is broadly conserved among apicomplexans [69]. The first clue as to the molecular identity of this nutrient pore came from the identification of PTEX [35], which suggested a potential function for EXP2 in formation of a membrane-spanning PVM pore involved in translocation of exported proteins into the erythrocyte cytosol. While the electrophysiological characterization of the nutrient pore [67] and the biochemical identification of EXP2 [70] were reported only 14 months apart, the relationship between these observations would not be realized for 25 years.

In contrast to other PTEX components which are restricted to the *Plasmodium* genus, EXP2 is broadly conserved among vacuole-dwelling apicomplexans. Disruption of the *Toxoplasma* EXP2 orthologs dense granule protein 17 (GRA17) and GRA23 alters the permeability of the PV (but not an analogous effector protein export pathway, in keeping with the lack of conservation of other PTEX components) [59]. While phenotypic analysis of EXP2 by conditional knockdown revealed the expected role in protein export, discordant EXP2 expression patterns relative to other PTEX components suggested roles beyond protein export [58,71]. Accordingly, the presence of the PVM nutrient channel, gauged by patch-clamp experiments, was found to depend on the abundance of EXP2. In contrast, inactivation of HSP101 did not impair detection of the nutrient channel, suggesting that EXP2 involvement in small molecule transport is independent from its export function [58]. Importantly, a carboxyl terminal truncation of EXP2 that removes a substantial number of charged residues while preserving essential function was found to alter the voltage response of the nutrient channel [58]. Alternation of the electrophysiological fingerprint of the channel due to a change in the EXP2 amino acid sequence shows that the nutrient channel is formed, fully or in part, by EXP2. This is reinforced by the observation that EXP2 forms a heptameric pore in the context of PTEX [33] and that recombinant EXP2, or a peptide comprising residues 61 to 96, can form pores in the absence of other PTEX components [72,73].

**Fig 4:**
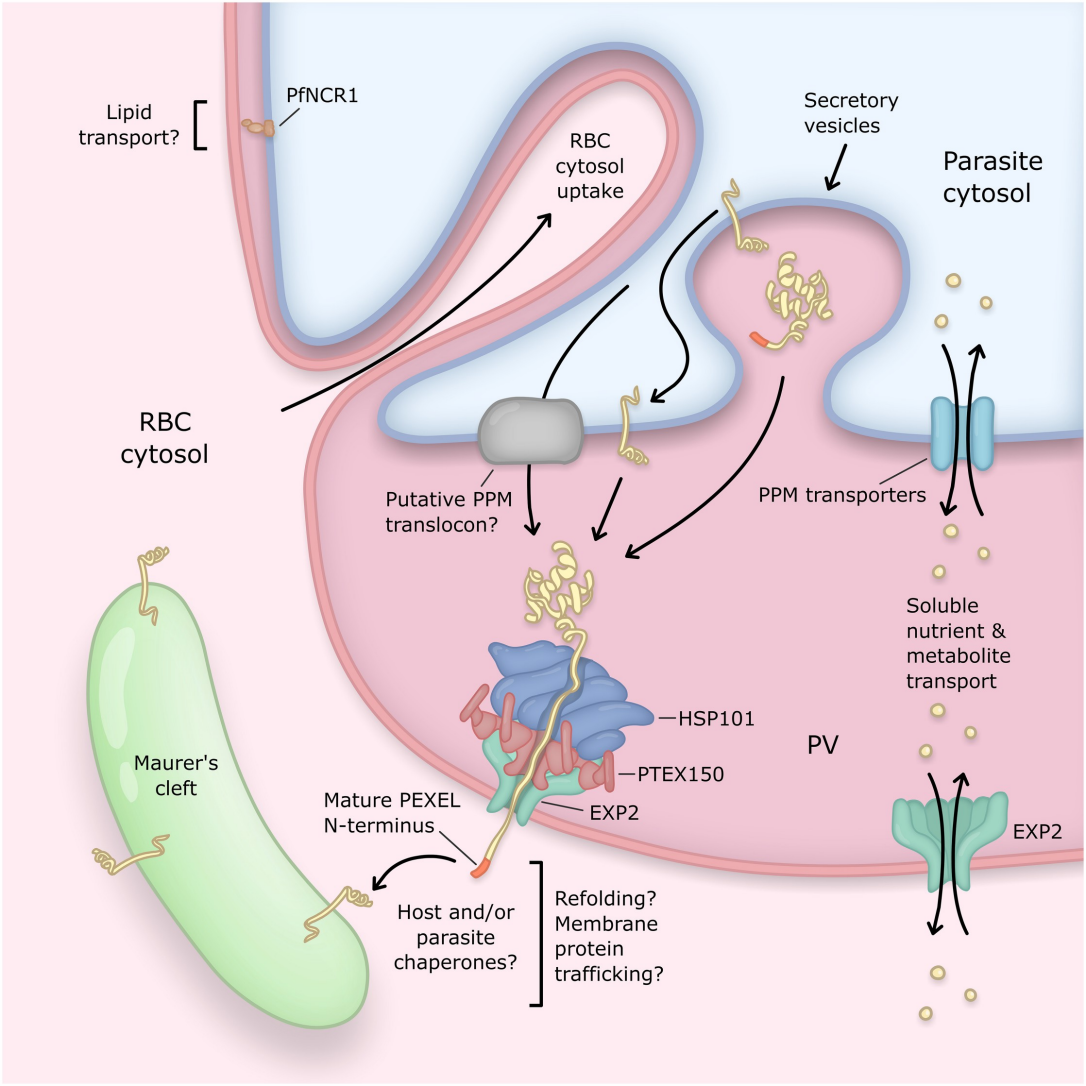
Transport activities at the PV of the blood-stage malaria parasite. The PV is the principal host–parasite interface and the site of several distinct transport activities mediating exchange of proteins and small molecules between the parasite and host compartments. The PV is compartmentalized into regions of close apposition between PVM–PPM (top left) and regions with greater luminal space (bottom right). Machinery involved in protein export and transport of solutes localizes to the latter regions. Parasite effector proteins destined for export into the host cell are delivered to the PV by secretory vesicles and then translocated across the PVM by the PTEX. Most exported proteins contain a PEXEL motif which is processed by the ER-localized aspartic protease Plasmepsin 5 to license export. Soluble exported proteins can be directly accessed by the PTEX AAA+ unfoldase HSP101 in the PV lumen, while exported integral membrane proteins are first incorporated into the PPM and then require a translocation step for PPM extraction that appears to involve unfolding power additional to HSP101. HSP101 is coupled to the membrane-spanning EXP2 pore via a flange-shaped adaptor known as PTEX150. Mechanisms for effector refolding, trafficking, and membrane insertion beyond the PVM are largely unknown and likely involve host and/or exported parasite chaperones. The EXP2 pore also serves a secondary role to transport small molecules across the PVM, likely independent of the PTEX complex. PfNCR1, a protein similar to the NPC1 protein involved in cholesterol egress from late endosomes, localizes to regions of close PVM–PPM apposition, suggesting that these may be sites of lipid transport. Parasites also endocytose large amounts of the erythrocyte cytosol through cytosomal invaginations of the PVM–PPM. ER, endoplasmic reticulum; EXP2, exported protein 2; HSP101, heat shock protein 101; NPC1, Niemann–Pick C1; PfNCR1, *P*. *falciparum* Niemann-Pick Type C1-related protein; PEXEL, *Plasmodium* EXport ELement; PPM, parasite plasma membrane; PTEX, *Plasmodium* Translocon of EXported proteins; PV, parasitophorous vacuole; PVM, PV membrane.

Interestingly, EXP2 is able to rescue the PV permeability defect incurred by *Toxoplasma* parasites lacking GRA17, suggesting that it can fulfill its nutrient transport function in the absence of other PTEX components or *Plasmodium*-specific cofactors [59]. Taken together with an apparent stoichiometric mismatch [58] and imperfect colocalization [74] between EXP2 and HSP101, this suggests a model where a PTEX-independent fraction of EXP2 comprises the PVM nutrient pore (Fig 4). While it is unknown if EXP2 oligomers adopt the same heptameric configuration in the absence of PTEX, electrophysiological and electron microscopy analysis of pores formed by recombinant EXP2 might suggest the existence of a larger oligomer of 10 to 12 subunits [72]. Additionally, an approximately 600- to 700-kDa complex containing EXP2, but generally not other PTEX components, is observed by blue native PAGE analysis of parasite extracts [34,36,75]. This is larger than expected for the EXP2 heptamer in isolation, which could represent a higher-order oligomer or additional cofactors that interact with PTEX-free EXP2 to support its function in nutrient transport. However, this might also reflect abnormal electrophoretic mobility (frequently observed for membrane proteins) or an artifact of detergent extraction.

One possible EXP2 cofactor is EXP1, a PVM protein that interacts with EXP2 and was recently found to be critical to parasite development but not protein export [76,77]. EXP1 is similar to the early transcribed membrane proteins (ETRAMPs), a group of charged, single-pass integral membrane proteins arranged into oligomeric arrays in the PVM [78,79]. Intriguingly, in parasites depleted of EXP1, the PVM nutrient channel could not be detected by patch clamp and parasites displayed hypersensitivity to nutrient starvation, indicating that EXP1 supports the small molecule functionality of EXP2 but not its role in PTEX [76]. Gross PVM abnormalities also occur in the absence of EXP1 and some PVM proteins, including EXP2 and certain ETRAMPs but not HSP101, cluster into loop-like protrusions [76,77]. Thus, while the influence of EXP1 on small molecule transport could represent a direct cofactor role required for EXP2 function (or the formation of a channel independent of EXP2), an alternative possibility is that redistribution of EXP2 may indirectly limit its transport capacity, for instance, by decreasing the PVM surface area accessible for transport. The function of the ETRAMPs is largely unknown, and it will be informative to determine if other members also contribute to PVM organization or transport.

Beyond parasite-encoded machinery, host factors may also be involved in PVM transport. For instance, aquaporin 3 (AQP3), a channel which transports water, glycerol, and other small neutral molecules, undergoes an apparent alternation in oligomeric state and is recruited to the parasite upon erythrocyte infection [80]. A selective inhibitor of AQP3 glycerol transport blocks parasite development, and it has been suggested that the channel may be functionally incorporated into the PVM [81,82]. Although independent patch-clamp studies have detected no additional PVM conductance beyond the EXP2-linked nutrient pore [58,67], AQP3 activity would not be apparent by this approach owing to its neutral substrate profile. Still, given the properties of the PVM nutrient pore, additional channels would seem superfluous, and clarity regarding the contribution of AQP3 at the PVM awaits alternative strategies to probe PVM transport.

### Additional small molecule transport mechanisms and PV compartmentation

The basis for uptake of sterols and the other lipids required by the parasite is less well understood. A curious inward cholesterol gradient is established in the iRBC [83], and the recent identification of PfNCR1, an ortholog of Niemann–Pick C1 (NPC1)-related proteins, in maintaining this lipid homeostasis has begun to shed light on this process [84]. In other eukaryotes, sterol transport is mediated by NPC1-related proteins and best understood in the lysosome where a partner NPC2 protein engages luminal cholesterol and passes it to NPC1, which forms a tunnel for transport across the glycocalyx and into the lysosomal membrane [85]. In *P*. *falciparum*, PfNCR1 localizes to the PPM with the extracellular domains oriented in the PV lumen. Inactivation of PfNCR1 renders the PPM hypersensitive to disruption by the pore-forming amphipathic glycoside saponin, indicating an alteration in the lipid content of this membrane and suggesting that PfNCR1 mediates transport of lipophilic molecules such as sterols or sphingolipids between the PPM and PVM to maintain low sterol content in the PPM [84].

Compartmentation of the PV is suggested by fluorescence recovery after photobleaching (FRAP) analysis with a fluorescent reporter targeted to the PV lumen, which may result from regions of differential spacing between the PVM and PPM [18,86]. Indeed, while the PVM and PPM are kept in close proximity during intraerythrocytic development, recent observations reveal that the PV is arranged into 2 domains defined by distinct spacing constraints between these membranes yielding regions of extremely close apposition resembling membrane contact sites (approximately 9-nm PVM center to PPM center) and regions with expanded PV lumen (20- to 40-nm PVM center to PPM center) [87]. EXP2 and a soluble PV lumen reporter both localize to the latter regions, indicating that these are sites of protein export and solute transport. In contrast, PfNCR1 appears to reside at the closely apposed regions of the PVM–PPM interface which anti-colocalize with EXP2. The positioning of PfNCR1 at these membrane contact sites could indicate direct transfer of lipids between PVM and PPM, although other proteins may also support the process, such as a steroidogenic acute regulatory (StAR)-related lipid transfer protein that localizes in part to the PV [88,89]. This bimodal domain organization indicates vacuole structure integrates lateral organization of PV proteins with vertical communication between the PVM and PPM to separate distinct types of transport. The factors that mediate PVM–PPM tethering and organization are currently unknown, although the disorganization observed following loss of EXP1 could suggest its involvement [76,77].

## Host cytosol uptake

During the course of intraerythrocytic development, the malaria parasite takes up vast quantities of the erythrocyte cytosol through an endocytic process that involves coordinated invagination of the PVM and PPM. These double-membrane structures, known as cytostomes and phagotrophs, appear to be the initiation site for an endolysosomal trafficking pathway that terminates in the acidic digestive vacuole [90]. Catabolism of hemoglobin, the principal component of the erythrocyte cytosol, within this compartment provides amino acids to support parasite metabolism (except isoleucine in humans, which is not present in adult human hemoglobin and must be taken up independently from the host [91]). However, most hemoglobin-derived amino acids are not directly utilized by the parasite and are instead released from the infected cell, a process that presumably requires efflux across the PVM via EXP2 [92,93]. Accordingly, this pathway is also important to free space within the host cell to allow for parasite expansion [94–96]. A byproduct of this effort is the liberation of enormous quantities of heme, which is polymerized into inert hemozoin crystals. The first molecular players in this trafficking process have recently been identified and suggest features reminiscent of clathrin-dependent and clathrin-independent endocytic systems in other eukaryotes (although clathrin itself appears uninvolved and restricted to Golgi trafficking functions in *Plasmodium*) [97,98]. In addition to its biological curiosities, this pathway is of great interest as it is the target of diverse antimalarials and alterations in the transport and trafficking machinery that support it underpin several mechanisms of drug resistance [99].

## Perspectives and future questions

### How are integral membrane proteins inserted into the PVM?

While recent insights into PTEX function provide a mechanistic model for translocation across the PVM, the basis for protein insertion into this membrane is not clear. A lateral gating capability in PTEX analogous to SEC61/Y, which introduces membrane proteins into a lipid bilayer by lateral release [100,101], is not suggested by the cryoEM structure [33]. Furthermore, knockdown of PTEX150 or inactivation of HSP101 does not impact insertion of EXP1 into the PVM, implying that membrane proteins are ferried from the PPM to the PVM by chaperones or vesicular transport [102]. In the case of EXP2, membrane insertion and/or oligomerization must be carefully controlled to avoid premature pore formation in the secretory pathway or PPM. By analogy, bacterial α-helix pore-forming toxins (α-PFTs) are maintained in a soluble, monomeric state that shields a hydrophobic transmembrane helix until interaction with components of the target membrane triggers conformational rearrangement, membrane insertion, and oligomerization [103]. While EXP2 is not structurally homologous to known α-PFTs [33], a similar mechanism could mediate PVM insertion, possibly sensing the elevated sterol content of this membrane relative to the PPM [83,104]. Membrane insertion and/or oligomerization might also be influenced by other factors such as a PVM insertase or interaction with PTEX150/HSP101 in the context of PTEX. Along these lines, rhoptry neck protein 3 (RON3), a multi-pass rhoptry membrane protein secreted into the PV during invasion, has recently been suggested to play a role in establishing proper EXP2/PTEX functionality [105]. Conditional deletion of RON3 leads to developmental arrest and death shortly after invasion that corresponds with defects in protein export and uptake of fluorescent glucose analogs, consistent with a function in establishment of transport activities at the PVM. Whether this contribution is direct or indirect awaits further elucidation of RON3 function and its resident membrane(s).

### How is exported cargo recognized by PTEX?

The signals that mediate cargo recognition by PTEX remain obscure, and it is currently unclear how the early events in the secretory pathway (PEXEL cleavage and acetylation) that appear to designate proteins for export are linked to cargo identification in the PV by PTEX, or whether they are linked at all. While the discovery of PEXEL processing by Plasmepsin 5 and subsequent N-acetylation suggest a possible PV export signal, these activities are not sufficient to activate export, which requires additional information immediately downstream of the mature PEXEL [10,27]. The nature of this information is obscure but transcends a recognizable linear motif and thus the role of PEXEL processing appears to lie in exposing this internal signal at the N-terminus of the mature protein.

PTEX cargo selection may be driven exclusively by HSP101, which contains an N-terminal domain (NTD) that is known to function in substrate recognition and hand off to the nucleotide-binding domains (NBDs) which power translocation through the central channel of the HSP100 hexamer (Fig 2H) [106,107]. HSP100 NTDs are variable in primary sequence, conferring plasticity in their binding capacity. Indeed, crystal structures of the NTDs from HSP101 and the related *P*. *falciparum* apicoplast chaperone ClpB1 reveal both adopt a common fold, but the HSP101 NTD shows substantial differences in surface electrostatic potential which may indicate a more promiscuous binding surface able to directly recognize structural features in the mature PEXEL/PNEP N-terminus [108]. Alternatively, cargo selection may also depend on other proteins that hand off exported substrates to HSP101, a common scenario for many HSP100s where the NTD works in conjunction with adaptor proteins or chaperones that can modify cargo specificity [109]. PTEX88, which interacts closely but dynamically with HSP101 [75], or other accessory factors might fulfill such a role to ferry certain proteins destined for export through the secretory pathway and/or PV.

The majority of PTEX150 is predicted to be disordered and was not resolved in the cryoEM structure [33]. These unresolved regions may serve additional functions beyond the role of PTEX150 as an adaptor connecting HSP101 to EXP2, possibly acting as a scaffolding for accessory proteins involved in cargo recognition or directly tethering exported proteins as they await translocation. Intriguingly, the PTEX cryoEM maps contain additional lower-resolution densities connected to the high-resolution regions corresponding to the well-resolved portion of PTEX150 (Fig 2G–2I). These form long helical densities that extend upwards from the end of each well-resolved PTEX150 protomer, terminating in short helical densities that form direct contacts with the HSP101 Middle domains (M-domains) [33], coiled-coil domains that encircle the first NBD ring. In homologs, the M-domains are known to play an important role in regulating the ATPase activity of the NBDs [110]. While the lower resolution of these additional densities prevented unambiguous assignment of their identities, an intriguing possibility is that they are formed by a portion of PTEX150.

Supporting this possibility, secondary structure predictions suggest that PTEX150 residues 838 to 873 form an unusually long helix matching the length of these long helical densities, which appear to be connected by an approximately 11 residue linker to the short helical densities (possibly residues 884 to 912 of PTEX150) that rest on top of the HSP101 M-domains. These short helical densities interact directly with aromatic residues at the midpoint of each M-domain. In HSP101 homologs, regulatory accessory proteins bind to aromatic residues on the M-domain to regulate ATPase activity of the HSP100 [111]. While endogenous PTEX150 could be truncated at residue 868 with no impact on parasite fitness, a truncation after residue 846, in the middle of the proposed claw helix, was unattainable, further suggesting an important function in this region [36]. If accurate, this would imply a role for PTEX150 in regulating HSP101 activity, possibly by controlling M-domain conformation, which is known to regulate ATPase activity in the related HSP100s [112].

### How are exported membrane proteins extracted from the parasite plasma membrane?

Many blood-stage exported effectors are integral membrane proteins and may require additional mechanisms to direct their trafficking through the intervening membranes that must be crossed to reach their ultimate destinations in the iRBC [10]. Single-pass transmembrane domain-containing exported proteins are inserted into the ER membrane and subsequently integrated into the PPM by vesicular traffic [27]. Intriguingly, addition of an unfoldable fusion close to the carboxyl terminal side the transmembrane domain results in the arrest of normally exported TM proteins at the PPM, indicating a translocation event is required for extraction from the PPM prior to subsequent translocation across the PVM (alternatively, increased spacing between the fusion and the transmembrane domain leads to arrest at the PVM) [27,30]. Given the close apposition of the PVM and PPM, HSP101 might also perform PPM extraction of these proteins. However, this model has been challenged by the serendipitous discovery that a bulky fusion on the HSP101 carboxyl terminus impairs translocation of soluble, structured proteins from the PV lumen [113]. Remarkably, this compromise in the export of structured cargo can be rescued by the addition of a transmembrane domain that produces a PPM-integral trafficking intermediate, suggesting that an unknown unfolding activity operating upstream of HSP101 is responsible for PPM extraction.

### How are exported proteins refolded and trafficked after crossing the PVM?

As PTEX and its associated machinery is located within the PV lumen and EXP2 does not extended beyond the surface of the PVM into the host cytosol, refolding and trafficking beyond the PVM is assumed to involve additional machinery [33,35,73]. In other translocon systems, chaperones on the trans surface of the membrane commonly participate in the translocation process (i.e., ER, mitochondrial and chloroplast import) [51]. Moreover, protein factors in the host cytosol have been found to be important for protein export in *P*. *falciparum* [31]. The mechanism by which exported integral membrane proteins are ultimately inserted into their target membrane system (Maurer’s clefts and host membrane) is also unclear but appears to involve a soluble intermediate step in the host cell [27]. One possible solution to this problem is the export of chaperones to support trafficking within the host cytosol. In *P*. *falciparum*, nearly half of the 49 parasite-encoded HSP40/DnaJ proteins are predicted for export, and some of these co-chaperones have been shown to interact with effector trafficking intermediates in the host cell [114–117]. An exported HSP70 known as PfHSP70x is also encoded by *P*. *falciparum* and involved in cytoadherence, but gene knockout and knockdown studies show that this chaperone is not broadly involved in export trafficking, nor is it conserved beyond *Plasmodium* spp. that infect great apes [114,118,119].

Given the many novel features of the trafficking pathway and membrane systems established within the iRBC, nonclassical parasite chaperones uniquely adapted to particular effectors may also function in this capacity. One emerging example is the RhopH complex, which is comprised of CLAG3, RhopH2, and RhopH3 and critical for establishment of PSAC at the iRBC membrane. In contrast to other exported effectors in *Plasmodium*, this initially soluble complex is stored in the rhoptries and injected into the PV during invasion before trafficking across the PVM and eventually to the erythrocyte membrane where CLAG3 is inserted as an integral membrane protein [120–123]. An RBC surface-exposed loop of CLAG3 is involved in modulating PSAC activity, suggesting that CLAG3 directly forms a novel type of anion channel [124,125]. Live-cell Förster resonance energy transfer (FRET) analysis shows that CLAG3 and RhopH2 remain associated throughout the entirety of their journey to the erythrocyte membrane [126]. Interestingly, loss of RhopH2 or 3 disrupts the complex and leads to rhoptry targeting defects, indicating a chaperone-like interdependence for proper trafficking through the parasite secretory pathway [122,127]. Recently, 2 independently determined cryoEM structures of a soluble form of the RhopH complex were reported, revealing that the predicted CLAG3 transmembrane helix is buried within the complex [123,128]. Thus, RhopH2 and 3 appear to represent a CLAG3-specific parasite chaperone system that maintains a soluble trafficking state until large-scale rearrangements enable CLAG3 insertion into the target membrane (RhopH2/3 also each contain a predicted transmembrane domain shielded in this complex and may also contribute directly to PSAC activity). Although conflicting results have been reported as to whether CLAG3 is dependent on HSP101 for localization to the host cell, both studies found that HSP101 activity is necessary for establishing PSAC activity [28,122], and it will be interesting to determine how this remarkable soluble complex transits the PVM.

Finally, hijacking of host chaperones may also serve parasite export trafficking in the erythrocyte cytosol. In support of this, the solubility of host HSP70 is altered in the iRBC [129], and yeast 2-hybrid assays indicate interactions between parasite-encoded HSP40s predicted for export and host HSP70 [130]. Additionally, the group II chaperonin TCP1 ring complex (TRiC) interacts with exported proteins in the host compartment [37]. While these observations raise intriguing possibilities, functional data clarifying post-PVM trafficking events are limited, and defining the mechanisms that facilitate effector trafficking within the host compartment remains an important area for future work.

### What mechanisms facilitate PVM transport in the *Plasmodium* liver-stage?

The vertebrate stage of malaria parasite infection begins with the injection of sporozoites during the bite of an infected mosquito. From the skin, sporozoites migrate to the liver where they invade hepatocytes and undergo a major increase in parasite biomass in which a single sporozoite generates thousands of erythrocyte-invasive merozoites which are then released into the circulation [131]. Although asymptomatic, parasites must pass through this developmental bottleneck to initiate the pathological blood-stage. Thus, the liver-stage is a major focus for therapeutic and vaccine development, partly owing to its suceptibility to a T cell–dependent sterilizing immunity [132]. While the relative ease of cultivating blood-stage *P*. *falciparum* has given rise to most of our understanding of PVM transport, the analogous processes at play during the less accessible hepatocyte infection remain largely unknown.

Malaria parasites are remarkable for their ability to develop in diverse erythrocyte and hepatocyte host cell environments that present distinct challenges and opportunities. Terminally differentiated erythrocytes lack both cell-autonomous defense mechanisms and the MHC antigen presentation system. Shielded within this environment, host cell remodeling during the blood-stage is focused on ensuring an adequate nutrient supply [5,61] and avoiding destruction by erythrocyte surveillance in the spleen that detects changes in iRBC rigidity and surface area [7,133]. By contrast, within the hepatocyte, parasites must evade cell autonomous innate immune mechanisms while preventing host cell destruction by apoptosis or immune surveillance [131,134,135]. Hepatocyte subversion involves manipulation of host gene expression and is expected to depend on a distinct repertoire of effector functionalities [136,137]; however, the identity of these effectors and their mechanism of export remain essentially unknown.

Several proteins are secreted into the liver-stage PV/PVM or the tubulovesicular network (TVN) [138] extending from it and are sometimes refered to as “exported” [139]. At present, however, only a few parasite proteins have been reported to pass beyond the PVM/TVN fully into the hepatocyte compartment, and in the present discussion, we reserve the term “export” exclusively for these. The first report of such a liver-stage “exported effector” was the major sporozoite surface protein known as circumsporozoite protein (CSP). CSP is critical to sporozoite motility as well as hepatocyte invasion and is the target of the RTS,S vaccine, making it a long-standing focus in sporozoite biology [140]. Curiously, this protein contains 2 PEXEL motifs and has been reported to localize to the cytoplasm and nucleus of hepatocytes infected with rodent malaria parasites [141,142]. This CSP “export” was found to be PEXEL-dependent and to alter host gene expression by virtue of a nuclear localization signal proposed to interfer with import of NF-κB to the nucleus [142]. However, subsequent studies showed that while hepatocyte presentation of CSP epitopes by MHC class I does require direct access to the cytosol, this process is not dependent on the CSP PEXEL motifs [143]. Furthermore, while Singh and colleagues found that the PEXEL-containing CSP N-terminus was able to mediate export of GFP in blood-stage parasites [142], Montagna and colleagues found that a similar fusion to ovalbumin did not pass beyond the TVN in liver-stage parasites [144]. At present, observation of CSP export during the *P*. *falciparum* liver-stage is lacking [145], and the nature and function of CSP trafficking within the infected hepatocyte remain unclear.

The second parasite protein reported to be exported into the hepatocyte is the Liver Specific Protein 2 (LISP2), a protein related to the *Plasmodium*-specific 6-Cys family [146] that localizes to the PV but also to the hepatocyte cytoplasm and nucleus during the later stages of liver-stage development [147,148]. Curiously, only an N-terminal portion of the protein appears to enter the host cell, while a carboxyl terminal fragment remains in the PV, suggesting that protein processing may be involved in LISP2 export [147]. LISP2 orthologs encoded by rodent malaria parasites contain multiple PEXEL or PEXEL-like motifs that are processed and N-terminally acetylated similar to exported proteins in the blood-stage [149], possibly explaining this phenomenon. In *P*. *falciparum* LISP2, only the most N-terminal of these is present and constitutes a “relaxed” PEXEL containing an additional residue between the third and fifth positions (RxLxxE/Q/D) [10]. While LISP2 is critical for late liver-stage development, it is unclear if this is tied to a function in the PV or the host cell [146,147]. In support of the latter, complementation with an N-terminal portion of the protein which corresponds to part of the region detected in the host cell partly rescues this defect [150].

**Fig 5:**
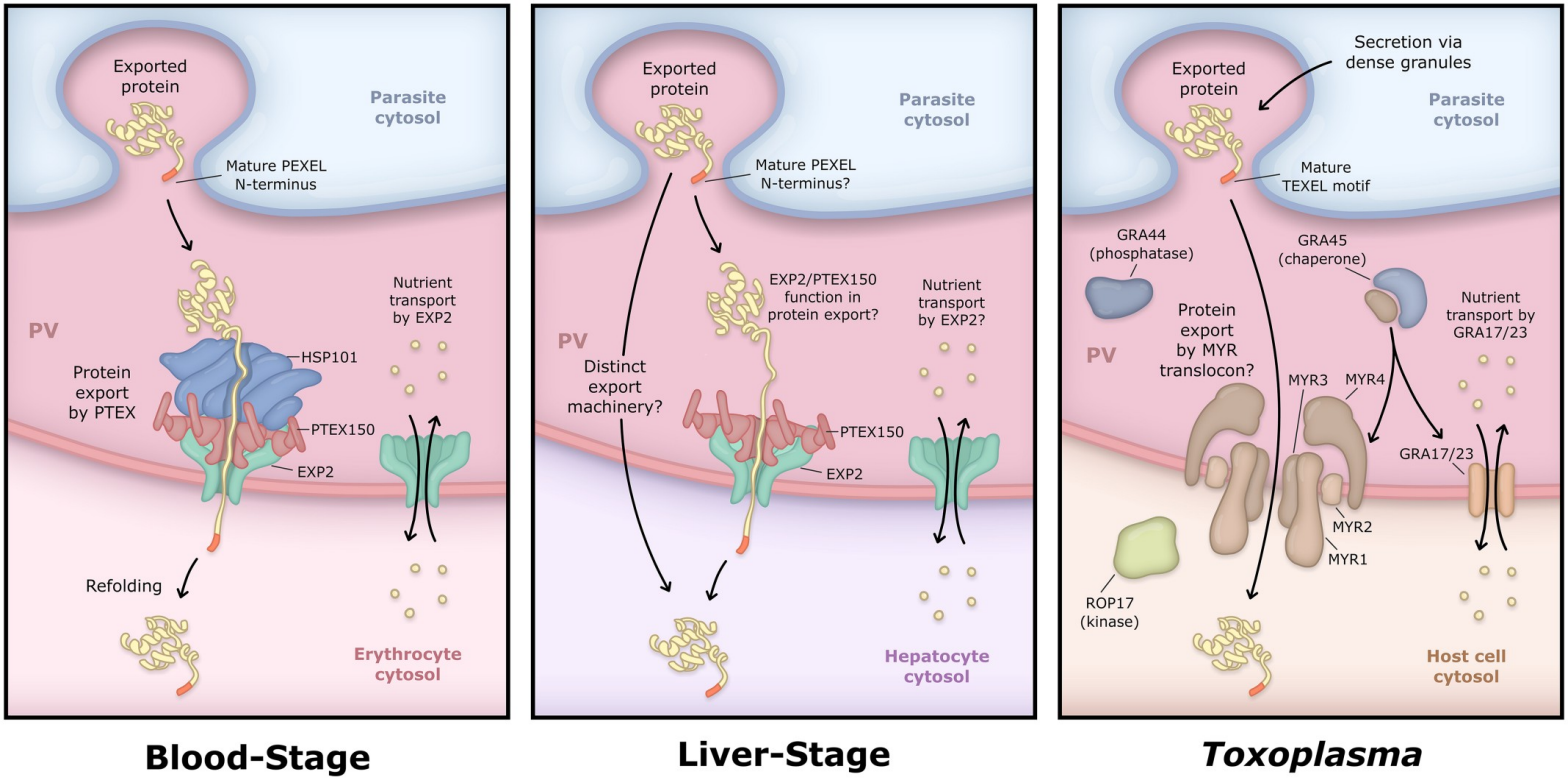
Comparison of PV transport in *Plasmodium* blood and liver-stages and *Toxoplasma*. In contrast to the blood-stage, HSP101 has not been detected in the liver-stage PV, suggesting fundamental differences in the export mechanism within the hepatocyte. While EXP2 has recently been implicated in sporozoite invasion of hepatocytes, it is unknown whether EXP2 and/or PTEX150 contribute to protein export or small molecule transport during intrahepatic development. While EXP2 orthologs GRA17 and 23 are required for small molecule transport across the *Toxoplasma* PVM, they are not involved in the export of dense granule effector proteins from the PV. Instead, a distinct set of proteins are required for export of these effectors including the membrane proteins MYR1–4 which are believed to form a novel translocon (possible MYR translocon organization shown here is purely speculative for purposes of illustration). Export also requires the putative phosphatase GRA44 and the chaperone GRA45, which may insert the MYR proteins into the PVM. Export additionally depends on the activity of ROP17, a kinase injected from the rhoptries into the host cytosol during invasion. ROP17 acts at the PV surface but the phosphorylated target(s) is not known. TgPPMC3, an additional phosphatase localized to the PV and involved in the export of a subset of effectors, is not represented in the cartoon. Processing of a TEXEL motif by the aspartic protease ASP5 is important for export of some *Toxoplasma* dense granule effectors while others lack the motif and are not processed by ASP5, although their export still requires ASP5 for unclear reasons. In contrast to the *Plasmodium* blood-stage, most ASP5 substrates are non-exported PV resident proteins, including MYR1, GRA44, and GRA45. EXP2, exported protein 2; HSP101, heat shock protein 101; PTEX, *Plasmodium* Translocon of EXported proteins; PV, parasitophorous vacuole; PVM, PV membrane; TEXEL, *Toxoplasma* EXport ELement.

While liver-stage exported proteins remain obscure, most such effectors would be expected to access the host cell shortly after invasion and should thus require an active translocation event to cross the intact PVM/TVN. Although PTEX was initially assumed to also constitute the liver-stage export machinery, subsequent observations have cast doubt on this model. Intriguingly, while PTEX core components EXP2 and PTEX150 are expressed in liver-stage parasites and localize to the parasite periphery (consistent with the PV), HSP101 could only be observed late in development, possibly corresponding to dense granule loading during merozoite formation [145,151,152]. This appears to indicate a fundamentally different mechanism of protein export and may explain the observation that proteins which are exported in the blood-stage accumulate in the PV/TVN and do not enter the host cell when expressed in the liver-stage [152,153] (Fig 5). Small molecule transport across the liver-stage PVM has been explored by dye permeability similar to *Toxoplasma* [154]. This study found a size-dependent molecular sieving activity at the the *Plasmodium berghei* liver-stage PVM with a cutoff of approximately 855 Da (Fura Red at 855 Da enters the PV while Calcium Orange at 1,070 Da does not), indicating that this pore might be distinct from the *Toxoplasma* or blood-stage *P*. *falciparum* pores (approximately 1,300 or approximately 1,400 Da cutoffs, respectively) [68,69]. On the host side, AQP3 is also recruited to the liver-stage PVM, paralleling observations in the blood-stage and again suggesting a possible alternative or additional transport mechanism. Strikingly, infected hepatocytes up-regulate AQP3 expression, and chemical inhibition as well as AQP3 knockdown are detrimental to liver-stage development, reinforcing an important role for this host factor in supporting the parasite [81,82].

Functional analysis of EXP2 in the liver-stage by a stage-specific conditional knockdown revealed EXP2 is important for transition from the sporozoite to the blood-stage, but it is unclear if this involves a function in protein export and/or small molecule transport within the hepatocyte [152]. Surprisingly, this stage transition defect was recently tied to an unexpected role for EXP2 in hepatocyte invasion [155]. In this study, extracellular sporozoites were shown to secrete EXP2 into the supernantant upon serum stimulation, and reexamination of the same conditional EXP2 knockdown parasites revealed a specific impact on invasion but not intracellular development. Strikingly, the invasion deficiency accompanying EXP2 knockdown was rescued in vitro by addition of recombinant EXP2 or the bacterial β-barrel pore-forming toxin α-hemolysin to the culture supernatant. This led the authors to propose a model where EXP2 secreted from extracellular sporozoites perforates the hepatocyte plasma membrane, leading to calcium flux and activation of host membrane repair pathways which mediate parasite invasion by an unknown mechanism. This prospect is further supported by the observation that knockdown of host acid sphingomyelinase, which is released from lysosomes to mediate membrane repair, hinders invasion, while recombinant acid sphingomyelinase added to the supernatant can also rescue the invasion defect. Remarkable, these findings reveal a distinct third function for the EXP2 pore beyond protein export and small molecule transport across the blood-stage PVM.

### PVM transport in related apicomplexans

The apparent nonessential role of EXP2 during intracellular liver-stage development [155] is suprising given that even if liver-stage protein export relies on a distinct translocon, EXP2 might still be expected to serve an important role in small molecule transport across the PVM. This would parallel the situation in *Toxoplasma*, where EXP2 orthologs GRA17 and GRA23 (the only PTEX-related components present; several additional HSP100s are encoded but are not found in the PV [156,157]) are only important for small molecule transport [59], while a distinct set of proteins is required for effector translocation into the host cell. Like *Plasmodium*, *Toxoplasma* effector delivery occurs via 2 distinct mechanisms: an initial bolus of effectors from the rhoptries is injected during invasion followed by secretion of a second group of effectors from the dense granules into the PV, which are then translocated into the host cell in a manner analogous to, but mechanistically distinct from, *Plasmodium* export [158]. PV-localized proteins required for *Toxoplasma* dense granule effector export include 4 novel membrane proteins known as MYR1–4 [159–161] and a putative acid phosphatase GRA44 [161,162]. While the specific functions of these proteins are unknown, MYR1–4 all possess predicted transmembrane domains and may form a novel translocon (Fig 5). An additional PV protein known as GRA45 is also required but appears to act broadly as a chaperone supporting dense granule secretion as well as insertion of PVM-resident membrane proteins such as MYR1-4 and thus its contribution to export is likely indirect [161,163]. Finally, ROP17, a kinase injected into the host cell from the rhoptries during invasion, is also necessary for export of dense granule effectors [164]. Additional MYR1-interacting PV proteins have been identified that are not strictly or broadly required for protein export [161] including TgPPMC3, a phosphatase important for efficient export of a subset of phosphorylated dense granule effectors including GRA16 and 28 [165]. Intriguingly, export of a phosphomimetic mutant version of GRA16 is similarly impaired, indicating that dephosphorylation of some exported cargo in the PV or dense granules is important for translocation into the host compartment [165]. These proteins are restricted to a subset of *Coccidians* and not conserved in *Plasmodium* spp., although the phosphatase domain of GRA44 bears some homology to UIS2, a PV-localized *Plasmodium* phosphatase important for blood and liver-stage development [166–168].

*Toxoplasma* exported dense granule proteins are intrinsically disordered [169–176] and fusion with a structured protein abrogates their export [160,177]. This is distinct from export in the *Plasmodium* blood-stage, where the unfolding requirement is less sensitive as cargo fusions to a structured fluorescent protein (GFP, etc.) are readily exported and only arrested by highly stablized folding states [30,32]. Curiously, substantial portions of both CSP and LISP2 are predicted to be unstructured, and liver-stage exported proteins might also be expected to require disorder given the lack of unfolding power without HSP101 [151,152]. The export power source for the *Toxoplasma* translocon is unclear and might be intrinsic to the MYR/GRA proteins involved or supplied by unknown PV machinery; alternatively, chaperones of parasite or host origin acting on the host side of the PVM could pull exported proteins across the vacuole. Indeed, the contribution of the ROP17 kinase occurs on the host cytosolic face of the PV, possibly involving the phosphorylation of transmembrane MYR proteins or other yet to be identified host or parasite factors [164].

While unique vacuolar translocation machinery may have developed between different apicomplexans, PEXEL-like proteolytic maturation in the secretory pathway is more widely conserved. In *Toxoplasma*, the Golgi-localized aspartic protease ASP5 is responsible for processing a PEXEL-related motif (RRL; known as the *Toxoplasma* EXport ELement or TEXEL) [177–179]. Similar to *Plasmodium*, cleavage of this motif in exported effectors is required for their translocation into the host cell [177]; however, in contrast to intraerythrocytic *Plasmodium* spp., most ASP5 substrates are non-exported PV resident proteins [180]. This includes the putative translocon components MYR1, GRA44, and GRA45, although the significance is unclear as processing of each of these proteins is dispensable for their function in export [161,162]. A more extreme example where PEXEL-like motif processing is dissociated from PV biology is provided by *Babesia* spp. These relatives of *Plasmodium* also infect erythrocytes but destroy the PVM moments after invasion [181], enabling protein secretion directly into the iRBC. While this obviates the need for a PVM translocon, *Babesia* exported effectors still contain a PEXEL-like motif which may mediate their packaging and release from secretory organelles [182,183].

## Conclusions

The PVM is the major line of demarcation between most apicomplexan parasites and their host cells. Nearly 30 years since the first transport activities were detected at this membrane [67], we have only just begun to understand the molecular mechanisms employed by these parasites to create and sustain their intracellular niche. The range of transport activities maintained at this membrane continue to attract enthusiastic study owing to the exciting new therapeutic targets that have been discovered at this site along with their fascinating biology. With emerging techniques such as single-particle cryoEM and cryo-electron tomography enabling unprecedented insight into challenging endogenous systems, we can look forward to an integrated view of the mechanisms employed by these master hijackers to communicate with and subvert their diverse host cells.
